# Why Do Students Feel Satisfied Yet Uneasy with Artificial Intelligence: A Process-Oriented Conceptual Review of How Cognitive and Moral Dissonance Account for the Satisfaction–Dissonance Paradox in Higher Education

**DOI:** 10.3390/bs16060846

**Published:** 2026-05-25

**Authors:** Debarshi Mukherjee, Lokesh Kumar Jena, Subhayan Chakraborty, Maidul Islam

**Affiliations:** 1 Department of Commerce & Business Studies, Jamia Millia Islamia, New Delhi 110025, India; dmukherjee@jmi.ac.in; 2 Department of Master of Business Administration, Gandhi Institute for Education and Technology (GIET), Khordha 752060, India; lokeshkumarjena@giet.edu.in; 3 State Panchayat Resource Centre (SPRC), Government of Tripura, Agartala 799003, India; subhayan.files@gmail.com; 4 International Business Department, Keimyung Adams College, Keimyung University, Daegu 42601, Republic of Korea

**Keywords:** AI, higher education, satisfaction, cognition, dissonance, PRISMA

## Abstract

The rapid integration of artificial intelligence in higher education positively affects student satisfaction, engagement, and learning outcomes. However, students frequently report ethical unease, guilt, and concerns about dependency. The current literature offers a limited explanation for their coexistence, as both have been treated as parallel or independent outcomes. Hence, this review extends and integrates existing theories by reconceptualising cognitive and moral dissonance as a central psychological process that explains how student satisfaction with AI-mediated learning is produced, negotiated, and sustained. Following PRISMA 2020 guidelines, we adopted a two-layer explanatory review design, synthesising 40 Scopus-indexed studies (Layer 1 = 15 studies; Layer 2 = 25 studies) from 2016 to 2025. Layer 1 studies explicitly define dissonance-related explanatory mechanisms that influence satisfaction and continued AI use across contexts such as dissertation writing, programming education, and problem-based learning. Layer 2 encompasses satisfaction-based studies that report ethical or affective concerns in parallel without theorising their interaction. The findings suggest a recurring satisfaction–dissonance paradox, in which students often experience genuine or conditional satisfaction from performance gains while simultaneously managing their psychological discomfort through one or more regulation mechanisms. Further, persistent and escalated dissonance leads to withdrawal or full or partial adaptive behaviour. We propose these dynamics as a testable Dual-Process Satisfaction–Dissonance Framework (DPSDF), which includes five dissonance triggers, five regulation strategies, three feedback loops, and four behavioural outcomes. Further, five domain experts’ suggestions have been taken to provide specific practical implications. This framework extends understanding of AI-mediated learning and provides foundations for future theory and policy development in higher education.

## 1. Introduction

AI is rapidly transforming the nuances of higher education, from intelligent tutoring and automated feedback systems to generative AI (Gen-AI) tools that support writing, problem-solving, and personalised learning (Basri, 2024; Cabeza-Rodríguez, 2025; Tang et al., 2025). The adoption of Gen-AI in education is diverse, including learning, dissertation writing, programming, training, and feedback systems. This reports on student satisfaction with increased efficiency, productivity, conceptual clarity, and perceived learning support (Anierobi et al., 2025; Subaveerapandiyan et al., 2025; Almufarreh, 2024; Alsulami et al., 2024). Further, students perceive improved academic performance with reduced cognitive workload (Almufarreh, 2024; Gökkurt Yilmaz et al., 2025; Tbaishat et al., 2025). However, students also report guilt, ethical unease, and discomfort related to integrity (Subaveerapandiyan et al., 2025; Tovmasyan, 2025). Particularly, the non-technical domain consistently raises concerns about originality, academic merit, and dependency, whereas the technical and problem-solving domains treat AI as a productivity tool with comparatively lower moral salience (Ren et al., 2025; Dawson et al., 2025; Gökkurt Yilmaz et al., 2025; Tang et al., 2025; Liu & Sun, 2025). It is true that students acknowledge the instrumental benefits of AI, as well as integrity, dependence, originality, fairness, and reduced concerns about independent learning (Chan, 2025; El Messaoudi et al., 2025; Ren et al., 2025). These concerns do not replace the satisfaction but rather create an ambivalence, which is repeatedly observed (Ngo et al., 2024; Subaveerapandiyan et al., 2025; Satoto et al., 2025; Farrokhnia et al., 2024). Hence, this contextual variation may not be understood by existing linear models alone, which require a mechanism to account for complex psychological processes shaped by task type, disciplinary norms, and assessment stakes.

Research on AI in higher education is largely outcome-oriented, synthesising determinants of satisfaction, technology adoption, usage intention, or performance. These approaches draw on the established Technology Acceptance and Expectation Confirmation Models (Alshammari & Babu, 2025; Ngo et al., 2024; Rodway & Schepman, 2023). Typically, these studies treat satisfaction and concerns as parallel outcomes yet offer limited explanation of their psychological interaction (Dorobăt & Corbea, 2025; Almufarreh, 2024). This leaves unexplained how students reconcile instrumental benefits with internalised academic values. Large-scale surveys report high satisfaction and continuance intention, whereas others document guilt, ambivalence, and restricted AI use in high-stakes academic tasks (Ren et al., 2025; Huang et al., 2025; El Messaoudi et al., 2025; Ngo et al., 2024). Hence, the literature remains fragmented, even though satisfaction and ethical concerns are widely documented and acknowledged.

Unlike earlier educational technologies, AI tools blur boundaries between assistance and authorship and actively participate in knowledge creation (El Messaoudi et al., 2025; Huang et al., 2025). The user acts passively as a moderator. Thus, these tools create conflicts between academic performance gains and values. For many students, this makes it unlikely that satisfaction is a direct response to usefulness or ease of use alone. It could be better conceptualised as a conditional, dynamic, evaluative state shaped by dissonance regulation and system qualities.

Therefore, this study addresses this gap by conceptualising cognitive and moral dissonance as an explanatory mechanism for student satisfaction in AI-mediated learning. This framework explains how student satisfaction with AI is produced, negotiated, and sustained, balancing academic gains against conflicts (Chan, 2025; Satoto et al., 2025). This review follows a two-layer design. Layer 1 explicitly or implicitly conceptualises dissonance-related processes that shape satisfaction, continued AI use, adjustment, or withdrawal behaviour (Chan, 2025; Ren et al., 2025; Satoto et al., 2025; Dawson et al., 2025; Hamid et al., 2023; Huang et al., 2025; Zviel-Girshin, 2024; Zhu et al., 2024), while Layer 2 synthesises only the parallel studies.

The following research questions are addressed:

**RQ1.**  *How does existing research conceptualise student satisfaction with AI in higher education*?

**RQ2.**  *How are ethical or affective concerns positioned in relation to satisfaction*?

**RQ3.**  *What forms of cognitive and moral dissonance have been identified in students’ use of AI*?

**RQ4.**  *How do these processes shape satisfaction and continued engagement and disengagement*?

### Contributions of This Study

This review makes four distinct contributions to the literature on AI in higher education. First, it reconceptualises student satisfaction as a negotiated and conditional psychological state rather than an evaluative endpoint. Second, it introduces a two-layer SLR approach that analytically separates the process and parallel studies from the broad satisfaction-based literature. Third, it extends existing theories and models in AI and higher education while not ignoring the necessary instrumental benefits, academic identity, and values. Lastly, it provides direction for future research by proposing the Dual-Process Satisfaction–Dissonance Framework (DPSDF), which offers testable propositions. Specifically, the DPSDF introduces three novel elements that are limited in existing TAM, ECM, UTAUT, or CDT models: (i) recursive feedback loops and a threshold assessment that model satisfaction as a dynamic and conditional state; (ii) a conceptually and theoretically derived closed set of five regulation strategies that directly link triggers to behavioural outcomes; (iii) a two-layer methodological template for separating descriptive from explanatory studies.

The remainder of this paper follows the theoretical and conceptual background, methodology, results, discussion and conclusion sections, respectively.

## 2. Theoretical Background

### 2.1. Limitations of Dominant AI Satisfaction Models in Higher Education

AI research in higher education has largely relied on the Technology Acceptance Model (TAM), Unified Theory of Acceptance and Use of Technology (UTAUT), Expectation–Confirmation Model (ECM), and related models (Davis, 1989; Venkatesh et al., 2003; Bhattacherjee, 2001). These models conceptualise satisfaction as a direct or mediated outcome of perceived usefulness, ease of use, system quality, and confirmation of expectations (Alshammari & Babu, 2025; Dorobăt & Corbea, 2025; Ngo et al., 2024). This is a coherent and internally consistent evaluative judgement. Meanwhile, ethical concerns, anxiety, and trust issues are typically treated as parallel constructs, moderators, or contextual limitations rather than as integral components of satisfaction. Hence, these linear acceptance models describe outcomes well but offer a limited understanding of how satisfaction and ethical concerns coexist psychologically.

### 2.2. Cognitive and Moral Dissonance in AI-Mediated Learning

Cognitive Dissonance Theory (CDT) provides an explanatory lens for individuals with inconsistent cognitions, i.e., conflicts between beliefs, values, and behaviours. It also explains how such individuals reduce this discomfort through cognitive or behavioural adjustment (Festinger, 1957; Harmon-Jones & Mills, 2019; Cooper, 2007). This concern is evident in the educational context, as students experience conflict between academic gains and values (Ren et al., 2025; Dawson et al., 2025; Satoto et al., 2025; Huang et al., 2025; Zviel-Girshin, 2024; Hamid et al., 2023). This does not mean leaving instrumental benefits behind for dissonance reduction but rather retaining them through rationalisation, justification, or selective reinterpretation to restore internal coherence. Thus, these patterns highlight that satisfaction is not merely an outcome of perceived usefulness but an effort to restore coherence between behaviour and values.

Contemporary CDT debates include whether dissonance is genuinely experienced or rationalised post hoc and how self-affirmation moderates dissonance reduction (Steele, 1988). The suggested framework adopts the experiential view but acknowledges that post hoc rationalisation may occur. Further, a related literature on technological ambivalence examines how users hold both positive and negative attitudes toward technology simultaneously (Mick & Fournier, 1998). Hence, the DPSDF extends this by specifying the psychological mechanisms (dissonance triggers and regulation strategies) that produce and manage such ambivalence.

In AI-mediated learning, dissonance arises when instrumental benefits, such as efficiency, productivity, and performance gains, conflict with internalised academic norms regarding effort, originality, autonomy, and merit (Chan, 2025; Ren et al., 2025). Hence, satisfaction is often not a sole response to utility or effectiveness; for many students, it emerges as a post-dissonance evaluative state.

This extends CDT into moral psychology within the educational domain, where behaviour threatens one’s moral self-concept (Blasi, 1984; Aquino & Reed, 2002). Students typically define themselves as primary authors, grounded in honesty, competence, and a sense of deserving achievement. However, these identities are now being challenged by AI tools that blur the boundaries between assistance and authorship (Chan, 2025; El Messaoudi et al., 2025). Students across domains have reported concerns about overdependence, skill erosion, authorship, and deservingness (Ren et al., 2025; Dawson et al., 2025; Gökkurt Yilmaz et al., 2025; Chen, 2025). These contextual modulations of satisfaction with AI vary with students’ efforts to preserve their moral and academic identity. This helps us understand students’ stable, fragile, or ambivalent satisfaction across different learning environments.

### 2.3. Dissonance Regulation, Self-Regulation, and Conditional Satisfaction

Individuals rarely ignore beneficial behaviour, even when experiencing psychological discomfort from dissonance, which they regulate through strategies such as restricting use and redefining the AI assistant as a moderator rather than an author (Chan, 2025; Ren et al., 2025; El Messaoudi et al., 2025; Satoto et al., 2025). Further, students must develop technical or algorithmic skills (i.e., prompt engineering) to improve efficiency, usefulness, and a sense of control and integrity (Ren et al., 2025; Zviel-Girshin, 2024; Hamid et al., 2023).

Therefore, satisfaction is not merely the absence of discomfort; rather, it is the successful management of discomfort through regulatory practices. This highlights that, when regulation is effective, satisfaction stabilises, whereas when regulation is ineffective, satisfaction becomes fragile or ambivalent or gives way to intermittent disengagement (Satoto et al., 2025; Subaveerapandiyan et al., 2025). Thus, conceptualising satisfaction is necessary, as it is an ongoing evaluative process that fluctuates with students’ perceptions of personal, moral, and institutional standards (Chan, 2025; Satoto et al., 2025; Huang et al., 2025). This moves beyond the dominant assumption of satisfaction as a stable endpoint.

### 2.4. Rationale for a Mechanism-Oriented, Two-Layer Review

Despite the theoretical relevance of cognitive and moral dissonance to satisfaction, existing literature has largely adopted aggregative, outcome-oriented approaches, synthesising determinants or prevalence of satisfaction. The mapping of satisfaction is appropriate and detailed, but it fails to capture psychological mechanisms. These mechanisms are unevenly theorised or implicitly embedded in empirical findings. Hence, this motivates an SLR focused on theory-building to distinguish between two analytically distinct bodies of literature, as existing models struggle to resolve this contradiction. This positions dissonance as an explanatory mechanism through which satisfaction with AI-mediated learning is produced, negotiated, and sustained.

### 2.5. Conceptualisation of Student Satisfaction

Across studies, student satisfaction is predominantly operationalised as a self-reported judgment or overall appraisal of AI-mediated learning, encompassing affective satisfaction, perceived learning satisfaction, and course satisfaction. This satisfaction mediates continuance intention (Almufarreh, 2024; Dorobăt & Corbea, 2025; Ngo et al., 2024). These assumptions are grounded in internal coherence, treating satisfaction as an outcome of perceived benefits, system quality, and ease of use (Alshammari & Babu, 2025; Rodway & Schepman, 2023). However, this review treats satisfaction with existing measures as a psychologically negotiated state rather than a unitary outcome, thereby managing cognitive and moral dissonance arising from AI use.

## 3. Methodology

### 3.1. Review Design and Rationale

This review follows PRISMA 2020 guidelines to ensure transparency, reproducibility, and methodological rigour in theory and concept development. This review employs the following two-layer analytical framework:

Layer 1: It includes studies that conceptualise cognitive and moral dissonance (internal psychological conflict) as an explanatory mechanism shaping satisfaction and continued behaviour.
Layer 2: These studies examine student satisfaction with AI while treating ethical, emotional, or integrity-related concerns as parallel outcomes, contextual variables, or limitations.

This two-layer approach integrates descriptive breadth and explanatory depth to address the contradictions in the literature, in which satisfaction and conflicts coexist through a process mechanism.

### 3.2. Data Source and Search Strategy

The Scopus database has been used to retrieve literature due to its broad, multidisciplinary indexing of journals and its search functionality (Falagas et al., 2008; Gusenbauer & Haddaway, 2020). We limited the search to Scopus due to institutional access restrictions. Moreover, as the objective was to define the recurring mechanisms rather than pooled effect sizes (e.g., meta-analysis), a single comprehensive database is considered sufficient. Further, we acknowledge that relying solely on Scopus may exclude relevant studies indexed in other databases, such as ERIC, PsycINFO, or Web of Science (Snyder, 2019). The search was conducted on studies from 2016 to 2025, reflecting the emergence and consolidation of AI in higher education.

The keyword search strategy combines terms that capture student satisfaction and dissonance-related psychological constructs. It includes AI, artificial intelligence, and generative AI, along with academic satisfaction, learning satisfaction, student satisfaction, cognitive dissonance, ethical dissonance, and moral conflict (see Table 1). Search terms did not include ‘ChatGPT’, ‘large language model’, ‘LLM’, ‘academic integrity’, ‘authorship’, ‘AI anxiety’, or ‘over-reliance’ because we prioritised broader psychological constructs. This may have excluded some relevant studies.

### 3.3. Eligibility Criteria

Peer-reviewed journal articles and review papers in final published form, written in English, and studies in higher education contexts have been included for synthesis. Conference papers, book chapters, editorials, non-final publications, studies in non-educational contexts, and AI without direct learner interaction, as well as ethical or integrity issues without linkage to student satisfaction, have been excluded. This synthesis includes only studies that discuss AI in relation to student satisfaction, engagement, evaluation, and learning experience.

### 3.4. Study Selection Process

**Figure 1:**
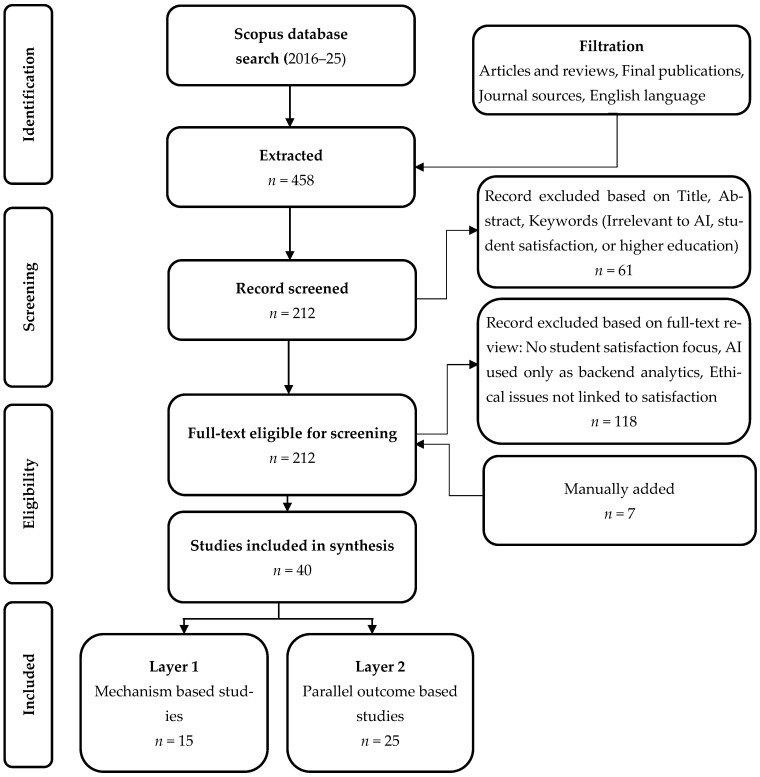
PRISMA flow diagram (Page et al., 2021).

As per PRISMA 2020 guidelines, 458 records were initially extracted and filtered by document type (articles and review papers), publication stage (final), source type (journal articles only), and language (English); 212 remained for title, abstract, and keyword screening. Following screening of titles, abstracts, and keywords, and a full-text eligibility assessment, 61 and 118 studies were excluded, respectively. A further seven studies were added manually through cross-referencing. As a result, 40 studies were retained for final synthesis. These studies were subsequently classified into the two-layer analytical framework, namely Layer 1 and Layer 2 studies (see Figure 1).

### 3.5. Two-Layer Analytical Classification

Three authors independently classified the 40 included studies into Layer 1 and Layer 2, and the fourth author resolved any disagreements in these classifications. Inter-coder agreement was 92.5% (37 of 40 studies). Disagreements on the remaining three studies were resolved through discussion with the fourth author. The two-layer classification was applied only after PRISMA inclusion to ensure screening decisions were not influenced by theoretical expectations. Layer 1 comprises 15 studies that explicitly or implicitly conceptualise internal psychological conflict as an intervening mechanism shaping student satisfaction, evaluation, or decisions about AI use. In Layer 2, 25 studies report satisfaction and ethical/affective concerns in parallel without theorising their interaction. This classification is analytical rather than hierarchical, distinguishing studies by their explanatory rather than descriptive focus. We did not use formal quality scores because this is a theory-building review focused on conceptual relevance to identify a dissonance trigger, regulation strategy, or satisfaction outcome.

### 3.6. Data Extraction and Synthesis

Literature has focused on educational context, student population, AI tools, reconceptualisation of satisfaction, and the role of ethical or affective concerns. Layer 1 studies were synthesised to develop the Dual-Process Satisfaction–Dissonance Framework (DPSDF), while Layer 2 studies were synthesised descriptively to identify existing patterns.

### 3.7. Methodological Rigour and Transparency

This study follows the PRISMA 2020 guidelines, which ensure transparency, replicability, and systematic reporting. Only peer-reviewed journal articles with conceptual relevance and explanatory contribution have been included and synthesised consistently for methodological rigour, rather than using a formal quality score. This is considered best practice for theory-building SLRS with explanatory depth (Snyder, 2019; Paul et al., 2024). Additionally, this study’s design and limitations have been acknowledged in subsequent sections for greater clarity and transparency.

## 4. Results

### 4.1. Dominant Parallel-Outcome Pattern (Layer 2)

Synthesis of Layer 2 studies consistently shows that student satisfaction with AI in higher education varies across geographies, disciplines, and methodologies (Almufarreh, 2024; Dorobăt & Corbea, 2025; Ngo et al., 2024; Rodway & Schepman, 2023; Tbaishat et al., 2025). Satisfaction is commonly measured through perceived usefulness, ease of use, system quality, or confirmation of expectations, which together drive continuous engagement.

Across studies, perceived usefulness, followed by ease of use of AI tools, is considered the dominant predictor across disciplines such as medical education, engineering, business, and the humanities. Further, confirmation of expectations based on ECM predicts satisfaction when expectations align with the actual experience.

Alongside satisfaction, substantial studies have also documented ethical concerns, anxiety, trust issues, dependency fears, or academic integrity risks (El Messaoudi et al., 2025; Huang et al., 2025). These concerns are almost uniformly treated as parallel outcomes, control variables, or contextual limitations rather than integrated into the satisfaction construct itself. Further, these studies treat both outcomes as independent phenomena that happen to co-occur, without theorising their psychological relationship. This parallel treatment persists across both qualitative and quantitative studies. However, few large-sample ECM studies have found a non-significant relationship between perceived usefulness and satisfaction (Ngo et al., 2024; Rodway & Schepman, 2023).

The coexistence of satisfaction and unease appears consistent across contexts and is shaped by infrastructure, economic, and cultural conditions. In resource-constrained settings, primary concerns centre on students’ accessibility and privacy. In transactional economies, it is associated with reduced human interaction and skill atrophy, raising concerns about authenticity. Similarly, in a technology-saturated environment, the concern is the perceived displacement of human judgement and academic labour. Furthermore, concerns vary by disciplinary norms. In the qualitative abstract domain, it rests on originality, authorship, and academic merit, which value individual voice and contribution. In the technical domain, it focuses on dependency and skill erosion rather than ethical issues (Ren et al., 2025; El Messaoudi et al., 2025; Dawson et al., 2025; Zviel-Girshin, 2024). In medical and health education, it lies in the middle, recognising the limitations of AI in clinical judgements and emphasising the accuracy of performance (Gökkurt Yilmaz et al., 2025; Chen, 2025).

Therefore, the Layer 2 studies provide a strong description of the parallel paradox but are limited by explanatory psychological mechanisms. Each study is summarised in Table 2.

Layer 2 studies show contradictions. For example, Ngo et al. (2024) found no significant direct path from perceived usefulness to satisfaction, whereas Alshammari and Babu (2025) and Dorobăt and Corbea (2025) reported strong positive effects. These discrepancies may stem from disciplinary differences (e.g., management vs. general samples) or measurement variance in satisfaction scales.

Disciplinary norms shape how concerns are reported. In medical and health education (Gökkurt Yilmaz et al., 2025; Chen, 2025), concerns centre on diagnostic accuracy and skill erosion. In the humanities and social sciences (El Messaoudi et al., 2025; Subaveerapandiyan et al., 2025), originality and authorship are paramount. In technical fields (e.g., Liu & Sun, 2025), ethical concerns are rarely reported, with AI treated as a productivity tool.

Most Layer 2 studies use cross-sectional, self-reported surveys with convenience samples. Only three studies (Tang et al., 2025; Gökkurt Yilmaz et al., 2025; Chen, 2025) used experimental or quasi-experimental designs. Common-method bias is likely, and causal claims are not supported. Satisfaction is consistently measured as a self-reported outcome, ethical concerns are documented but not integrated, and no study in Layer 2 uses dissonance as an explanatory mechanism.

### 4.2. Mechanism Patterns in Layer 1 Studies

These studies help develop process-oriented patterns in which satisfaction is actively established and maintained through regulatory strategies. Dissonance regulation spans dissertation writing (Ren et al., 2025), programming education (Dawson et al., 2025; Zviel-Girshin, 2024), coursework support (Huang et al., 2025), problem-based pharmacy learning (Hamid et al., 2023), and general academic use (Chan, 2025; Satoto et al., 2025; Zhu et al., 2024). This varies in intensity with assessment stakes and authorship expectations.

Initially, AI generates satisfaction through instrumental benefits, including greater efficiency and reduced workload (Chan, 2025; Ren et al., 2025; Satoto et al., 2025; Dawson et al., 2025; Hamid et al., 2023). Alongside satisfaction, moral or cognitive dissonance (conflict) arises between these instrumental benefits and internalised academic or personal values (e.g., effort, originality, autonomy, and fairness) (Chan, 2025; Ren et al., 2025; Satoto et al., 2025; Dawson et al., 2025). Based on the synthesis of Layer 1 studies, five types of dissonance triggers have been identified:

1. Value–behaviour conflict: contradiction between personal academic value and behaviour regarding effort and originality (Ren et al., 2025; Seran et al., 2025; Ren et al., 2026);
2. Expectation–reality gap: mismatch between anticipated and actual performance that causes frustration (Satoto et al., 2025; Zheng & Wang, 2026);
3. Ethical/perceived risk: concerns regarding privacy, bias, plagiarism, and academic integrity (Zhu et al., 2024; Chan, 2025; Alshamrani, 2026; Kirsanov et al., 2026);
4. Learning authenticity threat: genuine skill development concern (Dawson et al., 2025; Zviel-Girshin, 2024; Yang et al., 2026);
5. Peer/faculty judgment: others’ perception of AI use (Hamid et al., 2023; Huang et al., 2025; Kirsanov et al., 2026; Ren et al., 2026).

These triggers lead to cognitive or moral dissonance, either alone or in combination, depending on the type and intensity of the individual’s task demands and sensitivity.

Then, the students employ the five dissonance-regulation strategies as listed in Table 3 to manage their psychological discomforts. These five triggers and five strategies emerged from synthesis of the 15 Layer 1 studies. Further, alternative categorisations were tested but reduced clarity, and boundaries were validated by 92.5% inter-coder agreement (Section 3.5).

These strategies would help students realign AI use with their self-concept and stabilise satisfaction (Chan, 2025; Ren et al., 2025; Zheng & Wang, 2026; Oncioiu & Bularca, 2025). When regulation fails, students may experience fragile satisfaction, intermittent disengagement, or withdrawal from AI use (Ren et al., 2025; Huang et al., 2025; Satoto et al., 2025). The AI-guilt scale development study shows that guilt both mediates satisfaction and moderates continued AI use (Chan, 2025). Further, Zhu et al. (2024), Kirsanov et al. (2026), and Alshamrani (2026) found that ethical anxiety reduces AI use. Thus, Layer 1 studies define satisfaction not as a terminal outcome but as a negotiated evaluative state continuously shaped by regulatory strategies. Importantly, the four behavioural outcomes—harmonised satisfaction, fragile satisfaction, instrumental detachment, and conflicted dissatisfaction—do not appear verbatim in any single Layer 1 paper. Instead, they are theoretical syntheses of recurring empirical patterns observed across the 15 studies. The high-satisfaction, low-dissonance profile of harmonised satisfaction is reflected in Satoto et al. (2025), where Taiwanese academics converted AI frustration into exploratory recommitment, leading to continued high-intensity use and positive well-being. The high-satisfaction, high-dissonance profile of fragile satisfaction appears in Dawson et al. (2025), where students continued to use AI despite guilt and awareness. The low-satisfaction, high-dissonance profile of conflicted dissatisfaction is evident in Zhu et al. (2024), where AI ethical anxiety directly inhibited use behaviour, producing avoidant use and withdrawal risk. The low-satisfaction, low-dissonance profile of instrumental detachment is illustrated by Ren et al. (2025) and Zviel-Girshin (2024), where some students discontinued AI use or never adopted it due to ethical concerns, low perceived value, or successful substitution with other resources. Thus, while grounded in evidence, the naming and categorisation of these outcomes are conceptual contributions of this review, offering a clear typology for future research. Layer 1 studies have been summarised in Table 4, including the characteristics, context, and analytical explanation of the mechanism.

The following section (Section 5) synthesises these findings into a proposed framework and examines its theoretical and practical implications.

## 5. Discussion

**Figure 2:**
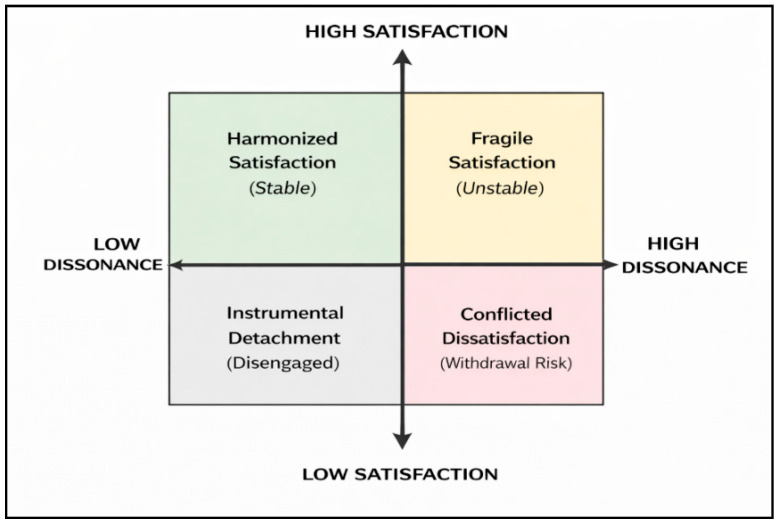
Satisfaction–dissonance state matrix in AI-mediated learning, Source: Authors.

**Figure 3:**
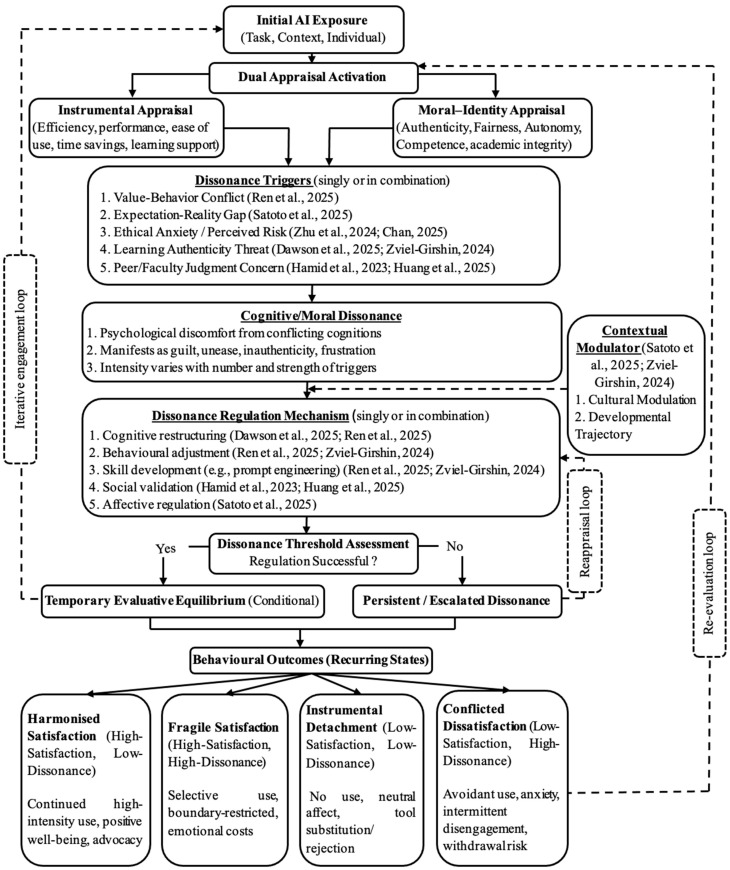
DPSDF framework for AI-mediated learning in higher education (Ren et al., 2025; Satoto et al., 2025; Zhu et al., 2024; Chan, 2025; Dawson et al., 2025; Zviel-Girshin, 2024; Hamid et al., 2023; Huang et al., 2025), Source: Authors.

In this section, we interpret the results by proposing the Satisfaction-dissonance Matrix (see Figure 2) and DPSDF (see Figure 3), and discussing its implications.

### 5.1. Integrating Layer 2 and Layer 1: Explaining the Satisfaction–Dissonance Paradox

The novelty of this review lies in integrating the descriptive breadth and explanatory depth of the Layer 1 and 2 studies, respectively. Layer 2 studies report that satisfaction with AI is widespread and resilient with ethical or affective concerns (Almufarreh, 2024; Dorobăt & Corbea, 2025; El Messaoudi et al., 2025; Ngo et al., 2024). The Layer 1 studies bridge the gap by addressing satisfaction as a negotiated state through active regulation. The comparison between the existing studies and this study is well detailed in Table 5.

The integration bridges gaps in the literature while acknowledging two alternative interpretations. First, discomfort may reflect social desirability rather than a genuine conflict, simply to appear principled. However, with consistent behavioural regulation, the impression can be managed (Ren et al., 2025; Satoto et al., 2025). Further, cross-cultural evidence suggests that students’ responses to AI-related frustration vary by context (e.g., cultural and infrastructure factors). For instance, in Indonesia, where switching costs are high, students continue to use AI out of inertia, even when frustrated. By contrast, in Taiwan, frustration leads to exploratory recommitment (Satoto et al., 2025). Second, satisfaction and dissonance may sometimes be sequenced, but recurring evidence supports ambivalence in ongoing negotiations. This contrast is clear: large-scale studies report uniformly high satisfaction with AI use, whereas mechanism-based studies deliberately limit or regulate its use to protect their academic identity. Based on this integration, we have proposed a Dual-Process Satisfaction–Dissonance Framework (DPSDF) that describes the whole mechanism.

To make the integration explicit, we map Layer 1 mechanisms onto corresponding Layer 2 patterns. By “mapping”, we mean theoretical alignment showing that the psychological processes identified in Layer 1 studies can coherently interpret the descriptive findings of Layer 2 studies. For example: the value–behaviour conflict (Ren et al., 2025) explains the coexistence of high satisfaction and plagiarism concerns (El Messaoudi et al., 2025); the expectation–reality gap (Satoto et al., 2025) accounts for the non-significant usefulness-satisfaction path in Ngo et al. (2024); skill development as a regulation strategy (Zviel-Girshin, 2024) corresponds to Basri’s (2024); and social validation (Hamid et al., 2023) mirrors Corbeil and Corbeil’s (2025) finding that dialogue and structure predict satisfaction. We emphasise that all mappings in this crosswalk are theoretically plausible alignments rather than empirically demonstrated causal relationships. The reviewed studies do not directly test the proposed mechanisms; they provide descriptive patterns that the DPSDF can coherently interpret.

### 5.2. Effectiveness of Dissonance Regulation Strategies

Table 6 details the dissonance-regulation strategies that shape satisfaction and continued AI use across three evaluative dimensions. Cognitive restructuring is the most crucial strategy for balancing AI use and academic identity. Sustained satisfaction without emotional cost can be achieved by reframing attitudes and AI as legitimate tools rather than threats (Dawson et al., 2025; Ren et al., 2025). By contrast, behavioural adjustment limits AI use to specific low-moral tasks. It is effective in the short run but does not fully resolve the issue if new task demands shift (Ren et al., 2025; Zviel-Girshin, 2024).

Similarly, developing skills in prompt engineering and verification practices is a promising strategy to increase satisfaction. Studies show that, over a semester, students with these skills report high satisfaction and confidence, which strengthens their sense of control and reduces dissonance (Ren et al., 2025; Zviel-Girshin, 2024). Social validation offers moderate benefits, but it is tempered by a diffusion of responsibility and fear of judgment. This is particularly prevalent among introverted students, and its effectiveness depends on supportive peer norms (Hamid et al., 2023). Lastly, affective regulation provides short-term relief but does not fully address the dissonance (Satoto et al., 2025). Thus, satisfaction is sustained through strategic regulation rather than genuine ethical resolution. Cognitive, behavioural, and skill-based strategies have been found effective in managing the sense of control and academic values.

### 5.3. Positioning the DPSDF Relative to TAM, UTAUT, and ECM

This review treats cognitive and moral dissonance as an intervening layer within technology acceptance models. TAM, UTAUT, and ECM explain initial adoption with perceived usefulness and ease of use; the DPSDF explains how satisfaction is sustained, destabilised, or renegotiated in AI-mediated learning contexts, shaping its durability rather than its emergence.

### 5.4. Interpreting the DPSDF

Figure 3 presents the DPSDF that reconceptualises dynamic student satisfaction with AI through a sequential process derived from Layer 1 studies, with descriptive patterns from Layer 2 used for contextual illustration. This also includes feedback loops with contextual modulations that shape students’ psychological behaviour.

#### 5.4.1. Initial Exposure and Dual Appraisal

The first step is initial exposure to AI use, which is shaped by task type, disciplinary norms, assessment stakes, and individual factors such as prior experience, AI literacy, and academic identity. This exposure leads students to engage in dual appraisal, i.e., instrumental and moral-identity appraisal. This duality occurs simultaneously and may lead to harmonious integration or psychological conflict. The instrumental appraisal drives initial satisfaction with efficiency, productivity, usefulness, ease of use, and learning support. Moral appraisal links AI use to self-concepts, including autonomy, authenticity, fairness, competence, and academic integrity (Chan, 2025; Ren et al., 2025).

#### 5.4.2. Dissonance Triggers and Cognitive and Moral Dissonance

The identified five dissonance triggers ignite cognitive and moral dissonance, either alone or in combination. The intensity depends on the number of triggers with students’ academic value system (Figure 3 and discussed in Section 4.2).

#### 5.4.3. Dissonance Regulation Mechanism

To manage and stabilise discomfort and satisfaction, students may choose various sole or combination regulation strategies out of five (see Figure 3 and Table 6).

#### 5.4.4. Threshold Assessment and Path Divergence

With regulation strategies, students undertake a dissonance threshold assessment. This helps them determine whether the chosen strategies have successfully managed their psychological discomfort. This follows two paths:

- If regulation is successful, students reach a temporary evaluative equilibrium, which is a conditional state in which satisfaction persists despite continued awareness of ethical tensions.
- If regulation fails, students experience persistent or escalating dissonance, as discomfort intensifies rather than resolves.

#### 5.4.5. Convergence on Behavioural Outcomes

The framework has a distinctive feature in that both successful regulation (negotiated equilibrium) and failed regulation (persistent dissonance) converge on the same behavioural outcomes. This further indicates that the response to AI use is not based solely on deterministic regulatory success and failure; instead, it reflects an interplay of perceived instrumental value, moral tolerance, and the strategies employed (Rodway & Schepman, 2023; Tbaishat et al., 2025). The DPSDF does not assume a deterministic mapping between regulation (success or failure) and behavioural outcomes. A student in temporary equilibrium may restrict AI use to maintain moral alignment, while a student with persistent dissonance may continue high-intensity use to justify past effort or because switching costs are high. Thus, the four behavioural outcomes are reachable from either regulatory path.

The interplay is well detailed in Figure 2 (satisfaction–dissonance state matrix). Although instrumental benefits are necessary for satisfaction, their durability remains insufficiently explained. Therefore, sustainability depends on moral and academic alignment through effective dissonance regulation, which varies over time and across contexts.

The framework acknowledges that satisfaction may persist even when regulation fails, provided compensatory engagement continues. Behavioural outcomes are not fixed but dynamic, shifting between states over time and across tasks. Thus, the relationship between regulatory success and behavioural outcomes is not deterministic but mediated by perceived instrumental value and moral tolerance.

#### 5.4.6. Feedback Loops

The feedback loops (see Table 7 and Figure 3) indicate that satisfaction with AI in higher education is a recursive process rather than a one-off adjustment. Thus, satisfaction is continuously recalibrated. Students reassess AI use across tasks and contexts and balance instrumental gain against academic value.

#### 5.4.7. Contextual Modulators

The framework also acknowledges that dissonance regulation is contextually shaped. First, cultural factors shape both choice and effectiveness, as evident in the study of Indonesia, where students continue to use AI despite high switching costs and institutional endorsement. Second, Taiwan’s technology-saturated environment prompts students to recommit by exploring alternative tools, which fosters engineering skills and transforms frustration into continued use and sustained satisfaction (Satoto et al., 2025; Zviel-Girshin, 2024).

### 5.5. Reconceptualising Student Satisfaction

In the context of AI-mediated learning, this review reconceptualises student satisfaction with AI use as conditional, dynamic and process-driven. Satisfaction begins with the initial instrumental benefit, whose durability aligns with how students regulate their dissonance to meet their academic and moral standards. This extends the dominant and continuance models by introducing the necessary internal psychological negotiations for sustained satisfaction. This pattern shows strong ethical sensitivity across disciplines and contexts, where satisfaction is driven primarily by adaptive feedback and performance support (Alsulami et al., 2024; Chen, 2025; Muthuswamy & Nithya, 2024; Cabeza-Rodríguez, 2025; Lv et al., 2025; Saqr et al., 2024).

### 5.6. Boundary Conditions and Scope of the Model

The framework is not uniform across AI-mediated learning, as dissonance regulation varies by task type, stakes, cultural context, institutional policies, and disciplinary norms (Ren et al., 2025; Huang et al., 2025). Intensive writing and assessment tasks (with strong norms and emphasis on originality) generate greater dissonance than low-stakes or exploratory learning practices (e.g., in computational or technical fields), where tool assistance is more normative (El Messaoudi et al., 2025; Chan, 2025; Gökkurt Yilmaz et al., 2025; Tang et al., 2025). With clear guidelines and transparent assessment design, dissonance may be regulated by reducing moral ambiguity, whereas weak or restrictive policies may intensify internal conflicts (Huang et al., 2025; Subaveerapandiyan et al., 2025). Thus, satisfaction is most fragile when instrumental benefits are high and clarity about normality is low.

#### Generalisability of the DPSDF

A majority of the 15 Layer 1 studies come from East Asian or non-Western contexts. The evidence base is regionally concentrated, so claims of universal applicability must be qualified. The framework should be tested in Western and other cultural contexts to support a better understanding of students’ satisfaction with AI-mediated learning in higher education and to substantiate strong generalisability claims. Layer 1 studies span three continents and six disciplines, whereas Layer 2 studies cover 16 countries across five continents. Despite this diversity, consistency has been observed across contexts in dissonance triggers, regulation strategies, and behavioural outcomes.

### 5.7. Theoretical Contributions

This review makes a strong contribution to theories that challenge the dominant assumption that student satisfaction with AI-mediated learning is a parallel outcome. It defines how satisfaction is constructed, regulated, and stabilised through negotiation with a central psychological mechanism. This further advances CDT in educational technology research by moving beyond linear models that balance academic identity and values. The mechanism reflects justification, self-regulation, and moral reconciliation in response to internal conflict, without ignoring core assumptions. It also introduces a two-layered analytical methodological SLR that separates explanatory depth from descriptive breadth for theory and conceptual development. Unlike TAM, UTAUT, and ECM, which treat satisfaction as a direct outcome of usefulness and ease of use, the DPSDF explains how satisfaction is sustained despite ethical concerns. Unlike CDT alone (which identifies dissonance but not specific regulation strategies in educational contexts), the DPSDF provides a closed set of five empirically observed strategies. The feedback loops and threshold assessment are absent in all prior models. The DPSDF is a conceptually and theoretically derived framework intended to organise existing evidence and generate testable hypotheses for generalisability. The DPSDF builds directly on Festinger’s (1957) core principles—conflict, discomfort, regulation, and restoration of coherence—but extends them by identifying context-specific triggers (e.g., value–behaviour conflict, expectation–reality gap) and regulation strategies (e.g., skill development, social validation) that are particularly salient in AI-mediated learning.

The DPSDF offers the following testable propositions for future empirical research: (P1) Under high-stakes assessment, cognitive restructuring leads to higher sustained satisfaction than affective regulation; (P2) students who develop prompt engineering skills report lower dissonance and higher continuance intention; (P3) when dissonance regulation fails, students may either withdraw from AI use or continue with persistent guilt and anxiety, in which continuation is more likely when perceived instrumental value and switching costs are high; (P4) clear institutional guidelines reduce moral ambiguity and weaken the value–behaviour conflict trigger.

Each trigger and regulation strategy in the DPSDF was derived from the Layer 1 studies (see Table 4 for DPSDF components). For example, ‘value–behaviour conflict’ was reported in Ren et al. (2025) and Dawson et al. (2025). ‘Skill development’ as a regulation strategy appears in Zviel-Girshin (2024) and Ren et al. (2025).

### 5.8. Practical Implications for Higher Education and AI Governance Derived from DPSDF

This study suggests that satisfaction is not an end product of instrumental benefits but rather the management of conflicts introduced by AI in higher education, with five trigger points. Thus, institutions must complement pedagogical and ethical support mechanisms for each technological innovation and its use (Huang et al., 2025; Satoto et al., 2025; Chan, 2025; Ren et al., 2025).

Effective and clear guidelines on AI use, transparent assessment design, and reflective engagement may help reduce moral ambiguity (El Messaoudi et al., 2025; Subaveerapandiyan et al., 2025). Educators may stabilise student satisfaction and responsible engagement by acknowledging ethical tension as a normal aspect of AI-mediated learning (Chan, 2025; Ren et al., 2025). Thus, transparent boundaries and reflective engagement may help mitigate these concerns.

Further, for the specificity of practical implications, suggestions (see Table 8) of five experts from the educational domain have been taken, which are logically derived from the DPSDF. These are not evidence-based best practices but testable interventions for future research and implementation. Each suggestion is directly linked to a specific component of the framework.

## 6. Limitations and Future Research

### 6.1. Limitations

This review has several limitations. First, the limited number of mechanism-based studies reflects the field’s emerging status. However, the conceptual constraint prioritises explanatory depth for theory development over numerical breadth. Second, this review spans different contexts without specific contextual comparisons. Third, it is limited to students’ satisfaction with AI use and does not consider the role of instructors or institutional actors in such a mechanism. Further, the exclusive reliance on Scopus, due to access restrictions to PsycINFO, ERIC, and Web of Science, may affect completeness. Thus, the Layer 1 typology (five triggers, five strategies) may underrepresent studies indexed in educational databases, such as ERIC and PsycINFO, especially those using alternative terminology (e.g., ‘academic misconduct’ or ‘AI anxiety’ rather than ‘cognitive dissonance’). The DPSDF is, therefore, provisional, pending broader database searches and empirical testing.

Similarly, studies rely heavily on cross-sectional, self-reported data. This raises common-method bias and precludes causal inference. Self-reported satisfaction may diverge from behavioural measures of continued AI use. Few studies employed experimental or longitudinal designs. Hence, to address these limitations, longitudinal designs and behavioural measures are needed to overcome the self-report and cross-sectional limitations of the current literature.

### 6.2. Directions for Future Research

Future research should focus on longitudinal and experimental designs to test the framework. Quantitative validation of the constructs of AI guilt, moral conflict, and regulatory strategies is required to strengthen the arguments. Further, comparative studies may help to set the boundary conditions. Additionally, the framework should consider how institutional policies, assessment design, and pedagogical framing would influence students’ ability to regulate dissonance and sustain satisfaction.

Similarly, as developmental trajectory helps regulate dissonance, a longitudinal design may help determine whether skill development provides a durable solution to dissonance or whether dissonance re-emerges with task complexity (Zviel-Girshin, 2024). Further, cross-cultural comparative studies may help to understand how institutional and cultural contexts shape regulation.

## 7. Conclusions

This review of 40 studies across countries and disciplines was synthesised using a two-layered analytical framework. We found that satisfaction with AI-mediated learning coexists with ethical unease. Satisfaction is not the outcome of instrumental benefits alone but a negotiated, conditional state shaped by the regulation of cognitive and moral dissonance. The proposed DPSDF suggests five dissonance triggers and regulation strategies, four recurring behavioural outcomes, and three feedback loops that capture the iterative and dynamic nature of the process. The DPSDF is a theory-building extension, not a replacement, of existing models. It integrates and proposes mechanisms that prior frameworks leave implicit. Further, the framework highlights the importance of contextual cultural modulation and developmental trajectory in effective regulation. This extends the conceptual model for future empirical research, offering testable propositions regarding the relationships among students’ instrumental benefits, academic value, and dissonance. Educators and policymakers may find this study useful for stabilising student satisfaction through stated mechanisms, with clear guidelines, scaffolding, and skill development.

## Figures and Tables

**Table 1:** Database search criteria, Source: Authors.

Databases	Keyword and Search Criteria	Date of Search
Scopus	(AI OR “Artificial Intelligence” OR “generative AI”) AND (“Academic Satisfaction” OR “Learning Satisfaction” OR “Student satisfaction” OR “Cognitive Dissonance” OR “Ethical Dissonance” OR “Moral Conflict”)	16 January 2026

**Table 2:** Layer 2: Summarised parallel-based studies, Source: Authors.

No.	Study	Educational Context	AI Application	Methodology	Analytical Framing of Satisfaction and Ethical/Affective Issues
1	Tbaishat et al. (2025)	UAE and Saudi Arabia (Zayed University, King Abdulaziz University)	Generative AI tools	Survey (PLS-SEM); *N* = 471	Satisfaction as the outcome of expected benefits, university support, ethical awareness, and technology self-efficacy mediated through behavioural intention. Ethical awareness was included as a predictor but was found to be non-significant; treated as a parallel without theorising its psychological interaction.
2	Althewini (2025)	Saudi Arabia (health sciences)	AI chatbots (academic advising)	Qualitative interviews; *N* = 4	Finds cultural and language sensitivity factors influencing satisfaction. Ethical/affective concerns are mentioned descriptively but not integrated into the explanatory framework.
3	Ahmat and Tang (2025)	Nursing education (international)	Generative AI chatbots	Literature review (8 studies)	Reviews evidence on AI chatbots in nursing education, finding improvements in knowledge, satisfaction, usability, and confidence. Ethical concerns are not examined, and satisfaction is treated as a direct outcome of AI functionality.
4	Anierobi et al. (2025)	Nigeria (Nnamdi Azikiwe University)	General AI tools	Survey (correlational); *N* = 631	Examines AI utilisation as a determinant of academic self-efficacy, engagement, and satisfaction. Satisfaction assumed internally coherent; ethical or affective concerns not examined.
5	Dorobăt and Corbea (2025)	Romania (IT students)	ChatGPT	Survey (SEM); *N* = 477	Perceived ease of use and perceived usefulness predict satisfaction and trust, which promote loyalty. Ethical concerns are not directly examined; satisfaction is treated as an outcome of cognitive and social perceptions.
6	Zhang et al. (2025)	China (programming)	AI-assisted learning (Programming Cat)	Survey (CFA, regression); *N* = 70	Assesses satisfaction across eight dimensions in AI-assisted learning. Finds learning sequence negatively correlated with satisfaction. Ethical concerns are not examined; satisfaction is treated as a multidimensional but internally coherent construct.
7	Tang et al. (2025)	China (middle school, Information Science)	Generative AI (SparkDesk)	Quasi-experimental, ANOVA (3 conditions); *N* = 131	Compares traditional teaching, AI-assisted without supervision, and AI-assisted with supervision. Finds AI with teacher supervision significantly improves engagement and knowledge mastery. Finds higher AI satisfaction with supervision and only for outcome-based. Ethical concerns are not directly examined.
8	Cabeza-Rodríguez (2025)	Spain (online university)	ChatGPT 3.5 assistant	Mixed-methods (EFA, CFA); *N* = 391	Finds efficiency is the most significant satisfaction factor. Accuracy and plagiarism concerns are reported qualitatively and not integrated as an explanatory mechanism.
9	Almufarreh (2024)	Saudi Arabia (University)	AI tools (general)	PLS-SEM-ANN; *N* = 355	Finds content quality, emotional well-being, perceived utility, and cognitive absorption as satisfaction predictors except perceived credibility. Lacks a mechanism interacting with satisfaction.
10	Basri (2024)	Saudi Arabia (university)	AI-powered tutoring systems	Survey (SEM); *N* = 284	Examines perceived usefulness, facilitating conditions, ease of use, and task value as predictors of satisfaction, engagement, and learning outcomes. Finds mediation and moderation effects among technical variables. Ethical concerns not examined; satisfaction treated as a technical outcome.
11	Gökkurt Yilmaz et al. (2025)	Turkey (dental students)	ChatGPT-4o with MeSH-based feedback	Randomised controlled trial; *N* = 110	Compares personalised AI-generated learning guides with traditional correct/incorrect feedback. The experimental group significantly outperforms the control in diagnostic performance and satisfaction. Ethical concerns were not examined. Satisfaction is treated as a direct outcome of feedback quality.
12	Liu and Sun (2025)	China (engineering, structural analysis)	AI-driven adaptive learning (fuzzy ELM)	Empirical study with fuzzy extreme learning machine	Develops an adaptive learning environment using fuzzy extreme learning machine and knowledge maps. Finds improved learning efficacy, resource integration, and knowledge mastery. Ethical concerns not examined; satisfaction treated as outcome of system performance.
13	Alsulami et al. (2024)	Saudi Arabia (Islamic University of Madinah)	AI-powered Quran reader (Maqraa)	Survey, *N* = 246	System quality and information quality predict usefulness and satisfaction, which predict performance. Finds significant interdependence between usefulness and satisfaction. Ethical concerns are not examined; satisfaction is treated as a mediator in the technical performance model.
14	Chen (2025)	China (medical students)	AI-driven personalised learning platform (Coze)	Randomised controlled trial; *N* = 40	Evaluates AI platform with dynamic learning path optimisation, affective computing, and intelligent resource recommendation. Technical efficacy focus; ethical concerns not examined. Satisfaction is treated as an outcome of platform features.
15	Saqr et al. (2024)	Saudi Arabia (University)	AI-driven e-learning platforms (Blackboard, Moodle, Edmodo, Coursera, edX)	Survey, *N* = 500	Tests integrated model with social learning networks, personal learning portfolios, and personal learning environments, influencing usefulness and ease of use, leading to satisfaction. Finds satisfaction predicts attitude but not intention. Ethical concerns not examined; satisfaction treated as a cognitive outcome.
16	Lv et al. (2025)	China (Shaanxi Normal University)	GenAI-supported MOOCs	Survey (SEM); *N* = 402	Test the learning experience framework with learning environment, teacher–student interaction, student–student interaction, and learning outcomes predicting satisfaction. Finds learning outcomes mediate the environment–satisfaction relationship; teacher–student interaction negatively affects satisfaction; student–student interaction is non-significant. Notes GenAI limitations descriptively but not as a mechanism.
17	Suchanek and Kralova (2025)	Czech Republic (management students)	ChatGPT	Survey (CB-SEM); *N* = 231	Finds job expectations and perceived quality predicting satisfaction. Ethical concerns not examined; satisfaction treated as outcome of expectation–performance comparison.
18	Tovmasyan (2025)	Armenia Uuniversity)	AI (ChatGPT) and digital technologies	Mixed-methods (interviews and focus groups); *N* = 200	Surveys students’ experiences with digital and AI tools. Finds 83.5% report improved academic performance; ChatGPT is widely adopted. Documents concerns about access, privacy, and reduced human interaction as challenges, but these are reported as parallel findings rather than integrated into satisfaction analysis.
19	Muthuswamy and Nithya (2024)	Saudi Arabia (University)	AI tools in mobile learning	Survey (PLS-SEM); *N* = 309	Tests the mediated-moderation model with visual learning style mediating the mobile learning interest–satisfaction relationship, and learner–instructor interaction and responsibility climate moderating. Finds all paths significant. Ethical dimensions are not examined despite a data-intensive platform; satisfaction is treated as an outcome of cognitive and social factors.
20	Corbeil and Corbeil (2025)	USA (graduate students)	AI chatbot	Survey, *N* = 47	Finds dialogue, structure, and autonomy significantly predict satisfaction and perceived achievement. Ethical concerns not examined; satisfaction treated as outcome of transactional distance dimensions.
21	Subaveerapandiyan et al. (2025)	Ethiopia (University)	AI chatbots	Survey; *N* = 367	Finds AI chatbots use moderate to high satisfaction levels. Documents concerns about access, privacy, content quality, ethics, and critical thinking loss as challenges. Provides recommendations for localisation and cultural sensitivity, but concerns are listed as parallel issues rather than integrated into the satisfaction model.
22	El Messaoudi et al. (2025)	Moroccan university	ChatGPT	Mixed-methods; *N* = 101	Finds satisfaction is strongly correlated with engagement. Documents concerns about accuracy, plagiarism, laziness, and skill erosion, which are reported in parallel with satisfaction.
23	Rodway and Schepman (2023)	UK (University)	AI educational applications	Survey, *N* = 302	Finds satisfaction drops when AI adoption is hypothetical. Discomfort is found with AI grading and well-being support. The psychological mechanism is missing.
24	Alshammari and Babu (2025)	Saudi Arabia (University of Hai)	ChatGPT	Survey (SEM); *N* = 297	Finds satisfaction mediates between usefulness, ease of use and behavioural intention. Ethical concerns are not examined, and satisfaction is treated as a mediator in the technology acceptance framework.
25	Ngo et al. (2024)	Vietnam (University)	ChatGPT	Survey (CFA, SEM); *N* = 435	Test the Expectation-Confirmation Model with expectation confirmation, perceived usefulness, satisfaction, and continuous usage. Finds all significant except perceived usefulness and satisfaction. Ethical concerns are not examined empirically.

**Table 3:** Dissonance regulation mechanism strategies, Source: Authors.

Strategy Family	Sub-Strategies	Studies
Cognitive restructuring	Attitude modification and downplaying limitations, along with considering AI as a tool, not an author	(Dawson et al., 2025; Ren et al., 2025; Seran et al., 2025; Alshamrani, 2026; Oncioiu & Bularca, 2025; Zheng & Wang, 2026; Ren et al., 2026)
Behavioural adjustment	Selective use and boundary-setting	(Ren et al., 2025; Zviel-Girshin, 2024; Hamid et al., 2023; Kirsanov et al., 2026; Zheng & Wang, 2026; Yang et al., 2026)
Skill development	Prompt engineering, domain-specific training, and verification practices	(Ren et al., 2025; Zviel-Girshin, 2024; Hamid et al., 2023; Seran et al., 2025; Yang et al., 2026; Zheng & Wang, 2026; Oncioiu & Bularca, 2025)
Social validation	Shared responsibility and peer comparison	(Hamid et al., 2023; Huang et al., 2025; Zheng & Wang, 2026; Kirsanov et al., 2026; Ren et al., 2026)
Affective regulation	Lowering expectation and frustration tolerance.	(Satoto et al., 2025; Zheng & Wang, 2026; Ren et al., 2026)

Note: the strategies are employed solely or in combination based on the type and intensity of the individuals’ task demand and sensitivity.

**Table 4:** Layer 1: Mechanism-based studies in conceptualising dissonance, Source: Authors.

No.	Study	Educational Context	Core Psychological Mechanism and Its Role	DPSDF Component(s) Identified
1	Ren et al. (2025)	Postgraduate (Master’s) students (China)	Satisfaction is sustained through justification, restricted use of AI, and domain-specific prompting. A feedback loop between cognition, emotion, and behavioural intention is found.	Value–behaviour conflict; Cognitive restructuring; Skill development
2	Dawson et al. (2025)	Undergraduate programming students (Germany)	Finds application-directed learners experience dissonance between high AI use and recognition, which undermines learning and satisfaction. Needed regulation through attitude modification or behavioural reduction for meaning-directed learning.	Value–behaviour conflict; Behavioural adjustment
3	Chan (2025)	Students (Hong Kong)	Finds moral dissonance (AI guilt) mediates satisfaction and moderates continued AI use.	Moral dissonance (AI guilt) as mediator/moderator for continued AI use
4	Satoto et al. (2025)	Academics (Indonesia and Taiwan)	Cultural modulation and developmental trajectory as regulation strategies for sustained satisfaction.	Expectation–reality gap; Affective regulation
5	Huang et al. (2025)	University students (China)	Finds ethical dissonance between the perceived prevalence and legitimacy of academic dishonesty, and ethical judgment may reduce such concern.	Ethical dissonance; Cognitive restructuring
6	Zviel-Girshin (2024)	Novice programming students (Israel)	Finds cognitive conflict between benefits and risks and prompt engineering skills as a regulatory strategy for improved satisfaction.	Cognitive conflict; Skill development
7	Hamid et al. (2023)	Pharmacy students (Malaysia)	Cognitive/ethical tension in collaborative learning; introverted students benefit from shared responsibility (reduced fear of judgment); risk of over-reliance weakening critical thinking; satisfaction coexists with reliability concerns; need for critical evaluation and cross-referencing.	Social validation; Learning authenticity threat
8	Zhu et al. (2024)	University students (China)	AI ethical anxiety directly inhibits use behaviour; perceived ethical risks influence use indirectly through anxiety; ethical awareness positively influences intention (control over controllable issues) but also increases perceived risk (uncontrollable issues); mediation pathways where ethical tension shapes actual use.	Ethical anxiety; Perceived risk
9	Seran et al. (2025)	University Student (Conceptual)	Explores how GenAI serves as both a trigger and amplifier of cognitive dissonance, creating psychological tension between AI-driven efficiency and principles of originality, effort, and intellectual ownership.	Value–behaviour conflict; Ethical anxiety/perceived risk; Skill development
10	Yang et al. (2026)	University student (China)	Investigates how GAI dependence drives three distinct skill adaptation pathways: deskilling (skill erosion), reskilling (acquiring new competencies), and upskilling (enhancing existing skills), which differentially impact learning outcomes based on task characteristics (substitutive vs. augmentative use).	Learning authenticity threat; Skill development and erosion
11	Ren et al. (2026)	Part-time university students (China)	Identifies five factors contributing to cognitive dissonance: competence challenge, relatedness gap, autonomy tension, value discrepancy, and role conflict. These reflect unmet psychological needs (SDT) and are shaped by socio-technological shifts, leading to the development of emotional regulation strategies.	Value–behaviour conflict; Identity concerns; Peer/faculty judgment
12	Zheng and Wang (2026)	University student (China)	Identifies three salient types of dissonance: efficiency-capacity dissonance, instrumental-traditional dissonance, and trust-reliance dissonance. Post-sort interviews further identify six self-regulation strategies: selective neglect, sequencing, reframing, context-based practice, verification, and conformity-based rationalisation.	Expectation–reality gap; Trust–reliance conflict; All 5 regulation strategies
13	Alshamrani (2026)	Systematic literature review across educational levels	A systematic review of psychological and ethical implications highlights concerns such as digital anxiety, overreliance, and ethical dilemmas concerning privacy, fairness, and transparency.	Ethical anxiety/perceived risk; Learning authenticity threat; Cognitive restructuring
14	Kirsanov et al. (2026)	University students (UK)	Investigates strategic non-disclosure of AI use, driven by fear of penalties and institutional ambiguity. Students use AI for supportive tasks (brainstorming, rewording) but rarely disclose due to perceived risk.	Ethical anxiety/perceived risk; Peer/faculty judgment; Behavioural adjustment
15	Oncioiu and Bularca (2025)	Universities students (Romania and Turkey)	Explores how knowledge-based strategies (knowledge valorisation, learning-oriented culture, active information sharing) in universities shape students’ legal and ethical literacy through the mediating role of AI governance and an ethical institutional culture.	Cognitive restructuring; Skill development; Institutional regulation

**Table 5:** Conceptual shift introduced by the present review, Source: Authors.

Dominant AI-in-Education Literature	Present Review
Satisfaction and dissonance are treated as parallel outcomes.	Dissonance is conceptualised as a psychological process shaping satisfaction.
Satisfaction is assumed to be stable and outcome-based.	Satisfaction is understood as negotiated and contingent.
Ethical concerns are framed as limitations.	Ethical tension is theorised as an explanatory mechanism.
AI benefits directly enhance satisfaction.	Conflict is regulated to sustain satisfaction.

**Table 6:** Effectiveness of dissonance regulation strategies in AI-mediated learning, Source: Authors.

Regulation Strategy	Dissonance Reduction	Satisfaction Preservation	Long-Term Sustainability	Supporting Studies
Cognitive restructuring	High	High	High	(Dawson et al., 2025; Ren et al., 2025)
Behavioural adjustment	Moderate	High	High	(Ren et al., 2025; Zviel-Girshin, 2024; Hamid et al., 2023)
Skill development	High	High	High	(Ren et al., 2025; Zviel-Girshin, 2024)
Social validation	Moderate	Moderate	Moderate	(Hamid et al., 2023; Huang et al., 2025)
Affective regulation	Low	Moderate	Low	(Satoto et al., 2025)

**Note:** The robustness of these strategies is further supported by recent studies: skill development (Yang et al., 2026; Zheng & Wang, 2026), cognitive restructuring (Oncioiu & Bularca, 2025), and behavioural adjustment (Kirsanov et al., 2026).

**Table 7:** Feedback loops in the DPSDF, Source: Authors.

Loop	Function
Reappraisal Loop	Allows students to attempt alternative strategies when initial regulation efforts fail
Re-evaluation Loop	Enables students to fundamentally reconsider AI’s instrumental value against moral-identity concerns
Iterative Engagement Loop	AI use with new tasks, changed contexts, or novel ethical considerations

**Table 8:** Expert recommendations derived from the DPSDF.

DPSDF Components	Expert Suggestions	Example Implementations
Value–behaviour conflict trigger	1. Pre-use reflective writing on academic values 2. Classroom discussions about AI and originality 3. Personal integrity pledges for AI use	1. “What does original work mean to you?” 2. Small-group debate: “Is using AI for outlines cheating?” 3. Acknowledgement: disclose all AI contributions
Expectation–reality gap trigger	1. Demonstrate AI errors before first use 2. Provide a comparison of AI vs. human output	1. Fabricated vs. original citations by AI 2. AI draft vs. without AI draft
Skill development regulation strategy	1. Prompt engineering workshops 2. Verification and fact-checking training 3. AI literacy micro-credentials	1. Hands-on writing effective prompts 2. Tutorial: using citation database to verify AI-generated references 3. Badge system: AI-assisted research certified
Cognitive restructuring regulation strategy	1. Reframe AI as “assistant”, not “author” 2. Use disclosure templates for assignments 3. Normalise AI as a tool	1. Syllabus language: You may consult AI as a writing assistant 2. I used ChatGPT for brainstorming but wrote all text myself 3. Compare to the calculator policy in math classes
Failed regulation	1. Low-stakes AI-integrated assignments 2. Ungraded practice with AI feedback loops 3. Peer review of AI-assisted drafts	1. Use AI, grade on reflection, not output 2. Try AI, then revise manually—no grade attached 3. Students share AI prompts and revisions in small groups

## Data Availability

Not applicable.
